# Geriatric Telemanagement of Health Conditions (GET HEALTH) in Nursing Home Residents Recently Discharged From the Hospital: Protocol for a Before-After Study

**DOI:** 10.2196/86001

**Published:** 2026-04-27

**Authors:** Giuseppina Dell'Aquila, Mirko Di Rosa, Irene Aguzzi, Francesca Suppa, Barbara Carrieri, Alessia Beccacece, Antonia Scrimieri, Lorena Rossi, Fabio Salvi, Massimiliano Fedecostante, Flavia Carle, Antonio Cherubini

**Affiliations:** 1 Geriatria, Accettazione geriatrica e Centro di ricerca per l’invecchiamento, IRCCS INRCA, Ancona, Italy; 2 Centre for Biostatistics and Applied Geriatric Clinical Epidemiology, IRCCS INRCA, Contrada Muoio Piccolo, Cosenza, 87100, Italy, 39 0718004604; 3 Center for Aging Technologies and TTO, IRCCS INRCA, Ancona, Italy; 4 Center of Epidemiology, Biostatistics and Medical Information Technology, Marche Polytechnic University, Ancona, Italy; 5 Regional Health Agency of Marche, Ancona, Italy; 6 Department of Clinical and Molecular Sciences, Marche Polytechnic University, Ancona, Italy

**Keywords:** telemedicine, televisit, nursing home, comprehensive geriatric assessment, geriatrician

## Abstract

**Background:**

Nursing home (NH) residents have a high burden of multimorbidity and disability and are frequently hospitalized. Comprehensive geriatric assessment might reduce hospitalization. However, few geriatricians are working in NHs. Technology might be used to overcome this problem.

**Objective:**

This study aims to verify if a geriatric telemanagement model improves patient outcomes in NH residents recently discharged from the hospital.

**Methods:**

Clinical outcomes of NH residents recently discharged from the hospital are compared before and after the implementation of GTM, where a geriatrician evaluates each patient during televisits with NH staff and discusses the case with the general practitioner. NH residents aged ≥70 years readmitted to NHs after hospitalization in the 3 years prior to the COVID-19 pandemic (2017‐2019) represent the control group enrolled in the retrospective preintervention phase. The primary outcome of the study is the hospital readmission rate during 6-month follow-up. Health technology assessment evaluates the cost-effectiveness of model implementation.

**Results:**

A total of 333 NH residents were included in the preintervention retrospective study. Data collection started in February 2022 and was completed in February 2023. A total of 104 NH residents were recruited in the intervention study, from March 2024 to March 2025. Data analysis is currently in progress.

**Conclusions:**

Currently, NHs in Italy are striving to manage the complexity of older patients who are often hospitalized. A geriatric telemanagement model might reduce hospitalizations, and therefore negative outcomes, as well as health care costs in this vulnerable population.

## Introduction

Nursing home (NH) residents are characterized by advanced age, multimorbidity, and a high burden of disability [1]. Hence, they often experience acute diseases or worsening of chronic diseases that might require hospitalization. In an Italian multicenter study, 11.6% of NH residents were hospitalized once during 1 year of follow-up [2]. Hospitalization might have a negative impact on NH residents, with increased risk of complications (ie, adverse drug reactions, delirium, falls, nosocomial infections, pressure sores), worsening health, and increasing disability [3,4]. Hospitalization is also associated with increased health care costs [5]. Moreover, NH residents discharged from the hospital have a higher risk of readmission [6]. Poor communication between hospital and NH staff, inaccurate discharge summaries, pending test results, incomplete medication reconciliation, and lack of follow-up might contribute to poor care transition and increase the risk of rehospitalization [7].

Several studies investigated the factors associated with hospital admission in NH residents. Both resident and NH characteristics have been implicated. Among the former, older age, male sex, and specific conditions (eg, infections, delirium, congestive heart failure, hip fracture, gastrointestinal bleeding, and disability) increased the probability of hospitalization, whereas longer length of stay, dementia, and terminal conditions decreased it [8,9]. Among facility characteristics, the lack of physicians during off hours might be one cause of inappropriate hospitalizations, as well as insufficient number of trained nursing staff and lack of the possibility of administering intravenous therapy [9,10]. On the other hand, a higher level of qualified staff can decrease this risk [2]. At least some hospitalizations from NHs have been considered potentially avoidable [9].

Several interventions have been implemented to reduce the hospitalization rate on NH residents. Among the most relevant studies, the Interventions to Reduce Acute Care Transfers (INTERACT) II is a quality improvement intervention that includes a set of tools and strategies designed to assist NH staff in early identification, assessment, communication, and documentation of changes in resident status. INTERACT II was evaluated in 25 NHs in 3 US states (Florida, Massachusetts, and New York) in a 6-month quality improvement initiative that provided tools, on-site education, and teleconferences every 2 weeks facilitated by an experienced nurse practitioner. During the study, there was a reduction in hospital admissions [11-13]. However, these results were not confirmed by a randomized implementation trial that compared changes in hospitalization and emergency department visit rates between the preintervention and postintervention periods in 85 NHs randomly assigned to receive training and implementation support on INTERACT or to a control group [14].

In a systematic review and meta-analysis, different transitional care interventions were shown to reduce rehospitalizations in older people living in NHs [15].

Among the included studies, 2 implemented a comprehensive geriatric assessment (CGA) to reduce hospitalization rate in NH residents, providing conflicting results [16,17]. CGA is a multidisciplinary evaluation in which the multiple problems of older persons are identified, described, and explained; their resources and strengths are assessed; the need for services is evaluated; and a coordinated plan of care is developed to address the identified problems [18]. CGA should be the most appropriate methodology of care for older NH residents [19].

However, a major obstacle to implement CGA in NHs in several countries, including Italy, is the dearth of geriatricians working in NHs. This problem could be, at least in part, overcome by means of technology. Long-term care is an area in which the adoption of technology might significantly improve health care. Telemedicine is feasible and effective for the delivery of specialist care to residents for selected medical disciplines. Gillespie et al [20] reported that in all intervention studies, except one, telemedicine use reduced hospitalization of NH residents up to 25%.

A recent cluster randomized clinical trial performed in French NHs found that a telemedicine CGA-based intervention significantly reduced the percentage of NH residents who experienced an unplanned hospitalization during 1 year [21].

The GET HEALTH (Geriatric Telemanagement of Health Conditions) study is a controlled before-after study aimed at verifying if a geriatric telemanagement (GTM) model using a geriatric televisit may reduce rehospitalization and improve clinical outcomes in NH residents recently discharged from the hospital. Moreover, the study aims at exploring the cost-effectiveness of the telemanagement model.

## Methods

### Overview

The GET HEALTH study is funded by the Italian Ministry of Health and the Marche Region within the multicenter project “Definition and testing of a new model of clinical governance based on the integration of tools such as Health Technology Assessment, Clinical Practice Guidelines, Clinical Pathways, and healthcare performance measurement for planning, implementation and management of healthcare interventions in different settings” (INTEGRATE-HEALTH-GOV; project code: NET-2018-12368077-4).

The GET HEALTH study is a controlled before-after study, consisting of both a retrospective and prospective component. The retrospective component of the study (ie, the control group) included NH residents of the Marche region who were readmitted in the NH after hospital discharge before the implementation of GTM. The prospective component (ie, the experimental group) included NH residents who were readmitted in the NH after hospital discharge and were managed with the GTM intervention in addition to usual care.

The GTM intervention was therefore compared with usual care, which consists of a general practitioner (GP) visit upon the patient returning to the facility after a hospital admission.

Inclusion criteria were permanent residence in an NH, aged ≥70 years, and at least 1 unplanned hospitalization during the study period. An additional inclusion criterion for the intervention phase was the ability to sign the informed consent form or the presence of a legally authorized representative who agreed to sign the informed consent form.

Exclusion criteria were temporary admission in the NH, emergency room access not followed by hospital admission, elective admission, and presence of a terminal condition.

In the original study protocol, the preintervention phase should have been carried out retrospectively in the 2 years before the implementation of the intervention of GTM. However, since the study officially started in September 2020 (ie, during the COVID-19 pandemic), this led to considerable postponement of its beginning. Moreover, the management of NH residents profoundly changed during the pandemic [22,23], with markedly reduced hospitalizations of NH residents and an increased rate of transfers among facilities [24].

In Italy, national and regional norms recommended that NH residents should be as much as possible cared within the NH, in order to reduce hospital admission and hence hospital overcrowding [25].

Therefore, it was decided to perform a retrospective analysis of outcomes and health care resource use among NH residents aged ≥70 years readmitted to NHs after an unplanned hospitalization during the 3 years preceding the COVID-19 pandemic (ie, from 2017 to 2019). Data collection was performed consulting the medical records stored in the facilities. Moreover, the regional databases of hospital admissions, emergency department visits, drug prescription, and health care utilization were consulted.

### Outcomes

The primary outcome of the study was the hospital readmission rate during the 6-month follow-up after hospital discharge. If a resident was rehospitalized more than once, only the first hospitalization was considered. The secondary outcomes were number of drugs, use of inappropriate drugs [26], incidence of pressure sores, falls, delirium in the 6 months after discharge, number of hospital days, number of emergency department visits, total number of hospitalizations, and the survival rate. Additional secondary outcomes for the intervention phase of the study were intervention adherence, NH staff perceived usefulness, and GP perceived usefulness.

### Intervention

The intervention consisted of 3 televisits (the first one within 1 week after hospital discharge, then at 1 month and at 3 months) where the geriatrician visited the patient and discussed each patient condition with NH staff and, in the context of the visit or at a separate time, with the GP.

The telemanagement system included a complete enterprise-grade videoconferencing system, and wireless videoconferencing carts were provided at each remote site to enable consultation at the bedside.

In the days before the first televisit, the NH staff uploaded the enrolled patient’s documents (discharge letter, medications taken upon discharge and prior to admission, and any other previous relevant information) to the platform to be reviewed by the geriatrician. During the televisit, the resident was assisted by a nurse who supported the interaction with the geriatrician, especially in case of severe hearing loss or cognitive impairment. The nurse provided medical information (ie, independence in activities of daily living [ADL], weight, height, presence of a bladder catheter, dysphagia or pressure sores) if the resident had dementia and also recorded vital signs, which were transmitted to the system in real time. Finally, the geriatrician, after collecting the patient’s personal, medical, and pharmacological history, performed the comprehensive multidimensional assessment (the 4AT test to detect delirium [27], the Short Portable Mental Status Questionnaire for the evaluation of cognitive functions [28], the 5-item Geriatric Depression Scale for the evaluation of depressive symptoms [29], the Index of ADL for the evaluation of ADL [30], and the Cumulative Illness Rating Scale for comorbidity [31]). The geriatrician then discussed the case by telephone with the GP, and afterwards drafted a report letter, always on the Telemedware platform. This letter was accessible at any time by the NH staff, and it was sent via a shared link, remaining available for 72 hours, for the GP. At subsequent televisits (1 and 3 months), the adherence (ie, the degree of implementation of the recommended interventions) was assessed using a specially designed form. The Index of ADL and 4AT scales were administered, the outcomes (hospitalizations, falls, delirium, and dysphagia) were verified, the current therapy and everything the patient had done since the previous televisits were reassessed, and finally a summary report was drawn up.

At the end of the 6 months, the project nurse collected all the information needed to measure outcomes (ie, falls, delirium, all tests and/or visits and/or hospitalizations performed since the last visit, any death) over the telephone. If the patient died before the end of the 6-month period, all the aforementioned information was collected at that date.

An online training course, lasting 6 hours, was developed to increase the geriatric knowledge of health care staff and GPs in the NHs involved in the project. The topics of this course included the following: the characteristics of older complex patients, multimorbidity, frailty, disability, geriatric syndromes (dementia, delirium, falls, instability, adverse reactions to drugs, immobilization syndrome), and CGA.

Furthermore, onsite technical training was carried out on the use of the technology made available to NHs. Each NH participating to the study received a technological kit consisting of the following equipment: a blood pressure and heart rate measuring device (FORA, Switzerland), a pulse oximeter (GIMA, Italy), a digital phonendoscope (Rudolf Riester GmbH, Germany); and a digital dynamometer for hand grip measurement (Kern, Germany). All the tools were certified as medical device class II and were connected via Bluetooth connection to a mobile device acting as a local hub. The data collection and transfer were managed through a telemedicine platform certified as medical device class I. The whole system was compliant with the General Data Protection Regulation (GDPR) and the service provider was certified according to ISO 13485, ISO 27001, ISO 27017, ISO 27018 and ISO 27701.

Finally, a structured questionnaire was administered to health care professionals involved in the prospective phase (ie, NH nurses and the GP) to assess the perceived usefulness of the new model. The questionnaire explores several dimensions: perceived clinical effectiveness, organizational impact, technological, training, and accessibility aspects. Moreover, it requested the health care professionals to compare the telemedicine model with usual care to highlight perceived advantages and weaknesses, which might differ by professional role. The objective was to gather useful information to evaluate the telemedicine care model used in the project. The questionnaire consisted of 38 questions divided into 5 sections that explored staff perceptions of the model’s implementation (eg, application of the model, effects on the patient care processes), the tools supporting the model (eg, technological context, training), ethics, and respect for patient data. The answers were categorized using a Likert scale, ranging from 1 (strongly disagree) to 5 (strongly agree). There was also the option “don’t know/not applicable.”

### Ethical Considerations

The study was approved by the IRCCS INRCA Ethics Committee (CE INRCA 19023 of 12.12.2019 for the preintervention study and CE INRCA 19024 of 12.12.2019 for the prospective intervention study). Due to the aforementioned reasons, an amendment was requested to modify the preintervention phase to include NH residents readmitted in the NH after hospitalization in the 3 years before the COVID-19 pandemic (2017‐2019). The amendment was approved by the ethics committee (CE INRCA 19023A of 28-1-2022) with a waiver for informed consent, in view of the time elapsed, considering that many NH patients died during the COVID-19 pandemic or were relocated. Moreover, the management of data was performed adhering to all the legal requirements, including the GDPR (EU) 2016/679.

### Assessment

Data collection for the preintervention retrospective study was carried out using an ad hoc form, with information concerning the index hospitalization (diagnoses of discharge, length of hospitalization, and drug therapy at discharge), the characteristics of the NH resident (diseases, functional status, state of the skin, presence or absence of swallowing problems, and drug therapy) 1 month before index admission, at NH return after hospital discharge, and at 6 months after the index admission. Furthermore, data concerning outcomes during the 6-month follow-up, such as rehospitalizations, emergency department admissions, falls, delirium, pressure sores, and death, were collected. Data collection was performed by trained research personnel consulting the clinical records and administrative data available in each NH.

The intervention study involved the evaluation of consecutive NH residents within 1 week of discharge from a hospital admission. The follow-up duration was 6 months. Data and information on hospitalizations (number and duration) and emergency room admissions not followed by hospitalization were collected. The number of medications taken, the use of inappropriate medications, and the incidence of bedsores, falls, and delirium were recorded at each follow-up and at the end of the 6-month period. Specifically, for medications, the current medication regimen on that day was considered. For delirium, falls, and bedsores, we investigated whether the patient experienced a new event since the last visit.

Moreover, a health technology assessment evaluated the effectiveness and the economic and social consequences of model implementation, by collecting data on health care resources consumption and costs both in the preintervention retrospective and intervention phases of the study. The economic sustainability of the model was evaluated using a cost-benefit analysis from the perspective of the National Health Service. This evaluation included both retrospective and prospective studies focusing on direct health care cost and reduction in health care resources used, which potentially result in the cost saving realized from the application of the model. The total cost was calculated considering the hours of the geriatrician who performed the teleconsultations (time spent conducting the teleconsultation, reviewing the patient’s documentation, drafting the summary document, and conducting a telephone conversation with the attending physician), the hours of the NH care staff who handled the teleconsultations (time spent by nurses conducting the teleconsultation), the hours of the project nurse to schedule appointments for televisits, and in some cases to carry out the televisit on behalf of the NH care staff when it was not available, and the hours used for staff training (online training course and time spent training staff to use the technology provided). The hours were recorded in an Excel timesheet. The cost estimation included also the fixed costs for the implementation of the telemedicine service (purchase and maintenance of the telemedicine platform and equipment).

The analysis employed a set of quantitative indicators to evaluate the economic impact of the project. Among these, the benefit-cost ratio was used to express the relationship between the total monetized benefits and the overall costs incurred. In this context, the benefits were estimated by quantifying in monetary terms the expected reduction in health care resource utilization, specifically the decrease in hospital admissions and emergency department visits attributable to the implementation of the proposed solution. Furthermore, the break-even point was calculated to determine the time horizon at which the cumulative benefits offset the initial and recurring costs, thereby identifying the point at which the intervention begins to yield net positive economic returns.

### Statistical Analysis

Descriptive statistics reported means and standard deviations or medians and interquartile ranges for continuous variables, as appropriate. Normality in distribution of continuous variables were assessed using the Shapiro-Wilk test. Absolute frequencies and percentages were presented for categorical variables. Comparison between groups (retrospective vs prospective study) for the primary endpoint (ie, at least 1 rehospitalization) was performed using unpaired *t* tests, Mann-Whitney *U* tests, or chi-square tests, as appropriate. Statistical significance for comparisons between baseline and follow-up was assessed via a paired *t* test or Wilcoxon matched pairs test for continuous variables and via a McNemar or Stuart-Maxwell test for categorical variables, as appropriate. Given the different levels of data, the statistical model took into account the existence of a clustered structure by NHs since multiple facilities were involved. Therefore, multilevel regression models adjusted for potential cofounders (ie, age, gender, number of diseases, number of medications, and impaired ADLs) were estimated in order to allow for the decomposition of total variability into a primary level (subject-related variability) and a secondary level (facility variability). Cox proportional hazard models were estimated to identify predictors of rehospitalization (primary endpoint) and mortality (secondary endpoint) and this assumption was tested using Schoenfeld residuals. Sequential imputation using chained equations with logistic regression methods were applied in case of missing values in covariates. Patients were followed until the end of the follow-up period, occurrence of the primary endpoint (rehospitalization), or death, whichever occurred first. Therefore, deceased participants were not excluded from the analysis. In order to ensure greater comparability between the 2 groups (retrospective and prospective), all analyses were repeated after propensity score matching, to reduce selection bias and balance baseline characteristics between 2 nonrandomized groups.

The sample size estimation for the preintervention retrospective study was based on the data of the Italian ULISSE study [2]. Considering that 8% of Italian NH residents are admitted to hospital once during 6 months of follow-up, and assuming a dropout rate of 20%, 245 subjects are enough to assure the same effect size with a power of 80% and alpha error of 0.05.

The sample size estimation for the intervention study was based on the review study by Gillespie et al [20]. In this review, all the studies, except one, showed that a telemedicine intervention reduced hospitalization rate, with percentages ranging from 4% to 25%. Therefore, in order to be conservative, assuming a reduction in the rehospitalization rate of 15% during 6 months following the telemedicine intervention and setting the statistical power at 90% and a significance level of 0.05, it was estimated through the calculation of proportions (1-tailed binomial test) that the expected sample size should be at least 93 subjects. Considering a drop-out rate of 10%, the estimated sample size increases to 102 subjects.

The G-Power 3.1 (Heinrich-Heine-Universität Düsseldorf, Düsseldorf, Germany) software was used to estimate this sample size. All the analyses were performed with SPSS 21.0 (IBM, Chicago, IL, USA). *P* values less than .05 (2-sided) were considered statistically significant.

## Results

For the preintervention retrospective study, 333 NH residents were included. The original preintervention study sample size was 245 older residents. However, during the study we decided to increase this number considering the fact that the need to enroll control NH residents retrospectively might have introduced differences in characteristics between the retrospective and prospective samples, since the older subjects included in the latter study had to sign an informed consent form, leading to the exclusion of those unable to provide it.

Data collection started in February 2022 and was completed in February 2023. For the intervention study, 104 NH residents were recruited from March 2024 to March 2025. Data analysis is in progress. The results will be published in peer-reviewed journals.

## Discussion

### Principal Findings

There is strong evidence that CGA improves outcomes in hospitalized older patients. CGA increases the likelihood of hospitalized older patients to be alive and in their own homes at 3 to 12 months after discharge [26]. However, the evidence is less clear in long-term care, although CGA is currently used in many NHs across the world. After the mandatory implementation of CGA in US NHs, improvement in several clinical outcomes of NH residents, improvement of care processes, and greater participation of health care professionals in team work were shown [32-35]. More recent studies confirm that NH residents may benefit from CGA in terms of improved quality of care although not necessarily experiencing a reduced risk of hospitalization. In a trial comparing a geriatrician-led outreach service to usual care for older residents discharged from the hospital (the intervention group received a postdischarge NH visit within 96 hours), there was a significant reduction in outpatient visits at 6 months in the intervention group but no difference in readmission rates [16].

At variance, another intervention study on recently hospitalized NH residents showed that the “Regular Early Assessment Post-Discharge (REAP)” protocol (first NH visit within 1 week after discharge and then monthly visits for 6 months by a geriatrician and a nurse practitioner) reduced hospital readmissions by two-thirds compared to usual care [17].

In Italy, the use of the CGA in NHs is limited, due to reimbursement issues and also to the insufficient number of geriatricians available. Telemedicine could fill this gap by connecting hospital-based geriatrician with the NHs.

The literature on the topic shows that the use of telemedicine in NHs is associated with a reduction in emergency room visits and hospitalizations of NH residents [19-37]. However, the comparison between studies is difficult because they differ in terms of methodology.

A 2010 pilot study in Taiwan [38] on a small sample of NH residents highlighted how a teleconsultation service with the hospital (mainly pulmonology, nephrology, orthopedics and physiatry consultations) can reduce hospitalization as well as nosocomial infections and adverse reactions to drugs. Hofmeyer et al (2016) [39] in a pilot study found that a telemedicine consultation service was associated with a decrease in hospital admissions during a 3-year follow-up in participating rural NHs, albeit the lack of a control group represents an important limitation of the intervention. In a randomized pre-post intervention study in 11 NHs in the United States, the hospitalization rate in the intervention group (n=700 NH residents) using telemedicine was reduced by 4.4% compared to the control group (n=1067) [40]. The intervention consisted of introducing into the NH a telemedicine service that allowed facilities to call primary care physicians after hours. In a retrospective study of 3510 patients in NHs or rehabilitation in the New York metropolitan area, the use of a virtual cardiovascular care program, consisting of an initial televisit, postconsultation care planning, and follow-up televisits, reduced the rate of rehospitalization due to cardiac disease (3% vs 10%, respectively) and all-cause (18% vs 27%, respectively) [41].

Recently, Gayot et al [21] performed a cluster randomized clinical trial in French NHs, showing that a telemedicine intervention implementing CGA significantly reduced the percentage of NH residents who experienced an unplanned hospitalization during 1 year, from 32% in the control group to 23% in the intervention group. However, the total number of hospitalizations was not significantly different between the 2 groups. The cost effectiveness analysis showed that the intervention reduced the hospitalization-related costs but did not produce an overall cost reduction. However, reducing the rate of admission to the hospital is still very important to avoid the risks of hospitalization in this vulnerable population.

### Conclusions

Currently, NHs in Italy are striving to manage the complexity of older patients who are often hospitalized. A GTM model might reduce hospitalizations, and therefore negative outcomes, as well as health care costs in this population. This study aims at verifying this hypothesis.

## Abbreviations

ADL: activities of daily living
CGA: comprehensive geriatric assessment
GDPR: General Data Protection Regulation
GET HEALTH: Geriatric Telemanagement of Health Conditions
GP: general practitioner
GTM: geriatric telemanagement
INTERACT: Interventions to Reduce Acute Care Transfers
NH: nursing home
